# Mast Cell–Tumor Interactions: Molecular Mechanisms of Recruitment, Intratumoral Communication and Potential Therapeutic Targets for Tumor Growth

**DOI:** 10.3390/cells11030349

**Published:** 2022-01-20

**Authors:** Deisy Segura-Villalobos, Itzel G. Ramírez-Moreno, Magnolia Martínez-Aguilar, Alfredo Ibarra-Sánchez, J. Omar Muñoz-Bello, Isabel Anaya-Rubio, Alejandro Padilla, Marina Macías-Silva, Marcela Lizano, Claudia González-Espinosa

**Affiliations:** 1 Departamento de Farmacobiología, Centro de Investigación y de Estudios Avanzados (Cinvestav), Unidad Sede Sur. Calzada de los Tenorios No. 235, Col. Granjas Coapa, Tlalpan, Mexico City 14330, Mexico; deisy.segura@cinvestav.mx (D.S.-V.); magnoliamarths@gmail.com (M.M.-A.); aibarra@cinvestav.mx (A.I.-S.); 2 Departamento de Inmunología, Instituto de Investigaciones Biomédicas, Universidad Nacional Autónoma de México, Ciudad Universitaria, Mexico City 04510, Mexico; itzel.irm@gmail.com; 3 Unidad de Investigación Biomédica en Cáncer, Instituto Nacional de Cancerología, Av. San Fernando No. 22, Col. Sección XVI, Tlalpan, Mexico City 14080, Mexico; omarmube@gmail.com (J.O.M.-B.); lizano@unam.mx (M.L.); 4 Departamento de Biología Celular y Desarrollo, Instituto de Fisiología Celular, Universidad Nacional Autónoma de México, Circuito Exterior S/N, Ciudad Universitaria, Mexico City 04510, Mexico; ianaya@ifc.unam.mx (I.A.-R.); mmacias@ifc.unam.mx (M.M.-S.); 5 Departamento de Microbiología y Parasitología, Facultad de Medicina, Universidad Nacional Autónoma de México, Circuito Exterior S/N, Ciudad Universitaria, Mexico City 04510, Mexico; padillaj@unam.mx; 6 Departamento de Medicina Genómica y Toxicología Ambiental, Instituto de Investigaciones Biomédicas, Universidad Nacional Autónoma de México, Circuito Exterior S/N, Ciudad Universitaria, Mexico City 04510, Mexico

**Keywords:** mast cells, cancer, signaling pathways, hypoxia, MC polarization, bioactive lipids

## Abstract

Mast cells (MCs) are tissue-resident immune cells that are important players in diseases associated with chronic inflammation such as cancer. Since MCs can infiltrate solid tumors and promote or limit tumor growth, a possible polarization of MCs to pro-tumoral or anti-tumoral phenotypes has been proposed and remains as a challenging research field. Here, we review the recent evidence regarding the complex relationship between MCs and tumor cells. In particular, we consider: (1) the multifaceted role of MCs on tumor growth suggested by histological analysis of tumor biopsies and studies performed in MC-deficient animal models; (2) the signaling pathways triggered by tumor-derived chemotactic mediators and bioactive lipids that promote MC migration and modulate their function inside tumors; (3) the possible phenotypic changes on MCs triggered by prevalent conditions in the tumor microenvironment (TME) such as hypoxia; (4) the signaling pathways that specifically lead to the production of angiogenic factors, mainly VEGF; and (5) the possible role of MCs on tumor fibrosis and metastasis. Finally, we discuss the novel literature on the molecular mechanisms potentially related to phenotypic changes that MCs undergo into the TME and some therapeutic strategies targeting MC activation to limit tumor growth.

## 1. Introduction

The immune system is composed of white blood cells, organs, and tissues of the lymphatic system, and diverse molecules such as antibodies, enzymes, and cytokines. Via a complicated network of processes and cells, the immune system detects injured tissue and recognizes self versus non-self, thus protecting the body from diseases of exogenous and endogenous origins and potentially dangerous molecules [1,2]. The relationship between the immune system and cancer development has been studied since long ago without being fully understood. For example, classical works conducted during the 17th and early 18th centuries demonstrated the relationship between immune response and tumor growth. One of them was undertaken by William Coley, who discovered that stimulation of the immune response by intratumoral injection of the *Streptococcus pyogenes* bacteria decreased tumor growth and burden in patients with sarcoma and melanoma [3]. Subsequently, Dr. Paul Ehrlich developed the concept of “immunosurveillance” to propose that tumors are constantly identified and eradicated by the host immune system even before clinical manifestations occur, thereby protecting the host from cancer [4]. Since the postulation of this hypothesis, many efforts have been made to elucidate the molecular mechanisms by which the immune cells can recognize and interact with tumor cells, contributing to the elimination, tolerance, or the expansion of the malignancy, with the hope of utilizing that knowledge to generate new therapies against cancer. 

Research showing that malignant tumors can also develop in patients with an apparent functional immune system indicates that immunosurveillance is not sufficient to explain the complex relationship between the immune system and malignant tumors. Those results gave rise to the postulation of a more complex and adequate term known as cancer immunoediting, a dynamic process that occurs in the development of tumors [5]. According to the cancer immunoediting hypothesis, the immune system may have a dual role in tumor progression, not only preventing but also shaping neoplastic disease [6]. Transformed cells survive and become more resistant to the elimination mechanisms, thus reaching a state that makes them resistant to immunosurveillance. Once that primary tumor is developed, both innate and adaptative immune cells can then turn into allies of tumors and promote a privileged environment where tumor growth continues unrestricted by immune pressure. Due to the complex relationship that exists between cell transformation, tumor growth, and immune detection, knowledge about the role of specific immune cell types in the distinct phases of cancer immunoediting becomes critical to understand how tumor cells are detected by the immune system, and how the ability of the immune system to fight against cancer could be restored. 

Tumor-associated immune cells are the main players in cancer immunoediting. In particular, innate immune cells, such as macrophages, antigen-presenting dendritic cells, or mast cells have been associated with both pro-and anti-tumoral responses. Those findings have opened up new insights regarding therapeutics against cancer. In this review, we will discuss some of the main aspects of the relationship between MCs and tumors, focusing on the mediators and signaling pathways involved in the chemical communication between tumor cells and MCs that lead to MC recruitment, their effects on angiogenesis, and other intratumoral processes that lead to tumor growth and, finally, the application of that knowledge to the design of MC-directed therapeutic strategies to control cancer development. 

## 2. The Multifaceted Role of Mast Cells in Solid Tumors 

**Figure 1:**
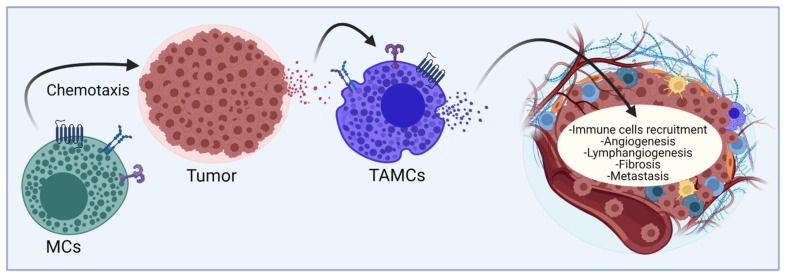
Current vision of the relationship between MCs and malignant tumors. Tissue-resident MCs and probably MCs precursors are incorporated to the sites of tumor growth through the action of distinct chemokines and active lipids. Under the influence of the TME, MCs modify their phenotype towards TAMCs and secrete mediators that contribute to the generation of new blood and lymphatic vessels, the recruitment of distinct immune cell lineages and the fibrosis of tumor mass. Mediators can also promote metastasis of primary tumors. The final consequences of MCs’ activation can be considered pro-or anti-tumorigenic, depending on still undefined conditions. Figure made using BioRender, accessed on 7 January 2022, agreement number HV23G71UG7.

Mast cells (MCs) are tissue-resident elements that translate stimuli from the local environment into the controlled secretion of active chemical mediators through the activation of specific signaling pathways connected to different secretion pathways [7]. Those mediators modulate host-protective immune responses against bacteria, parasites, and viruses, but are also associated with a detrimental role in allergic diseases and cancer [8,9,10]. It is well known that MCs can infiltrate many solid tumors and, once there, they are known as tumor-associated MCs (TAMCs) (Figure 1) [11]. TAMCs are considered distinguishable participants and orchestrators of both pro-tumoral and anti-tumoral responses and represent one of the most controversial immune cell types in cancer since on one side, they can promote different processes that lead to tumor progression such as angiogenesis, lymphangiogenesis, fibrosis, and metastasis, but, in addition, TAMCs can release mediators capable of inducing the recruitment of other immune cells into the tumor that can perform pro- or anti-tumor functions (Figure 1). It is now accepted that a complex interaction between tumor cells and MCs exists and needs to be closely analyzed.

The role of MCs in cancer has been addressed utilizing distinct experimental approaches, such as the histological analysis of MC distributions in animal and human solid tumors (reviewed in [11,12]). In those works, the capacity of MCs to infiltrate the solid tumor has been considered an important marker associated with either better or worse prognosis depending on the type and tumor stage. In some solid tumors, MCs are detected in the intratumoral areas, while in others they are preferentially located in the peritumoral zones. Intriguingly, the presence of peritumoral MCs seems to indicate bad prognosis, while its intratumoral location is associated with both favorable and unfavorable prognoses (Table 1). Some studies have made efforts to associate the presence of TAMCs in peritumoral or intratumoral areas with specific tumor-associated processes. For example, in lung cancer, there is a positive correlation between MCs’ numbers (identified as tryptase and chymase positive) and microvascular density in the intratumoral zone, supporting the involvement of MCs in the angiogenic process [13]. In some types of tumors, MCs’ infiltration correlates with tumor progression, invasion and increased microvessel density, whereas in others, the correlation is the opposite [12].

Other strategies to address the role of MCs in tumor growth are based on the use of MC-deficient mice. Utilizing approaches such as the inoculation of transformed cells, tumorigenesis induced by mutagen exposure, or crosses with genetically modified animals that produce distinct types of malignant tumors, researchers have taken advantage of some mice whose mutations and genetic characteristics provoke the lack of MCs. In general, tumor size, metastatic capacity, blood supply, lymphatic vessel generation, and tumor fibrosis are analyzed in MC-deficient animals and the role of MCs is confirmed by the reconstitution of MC-deficient mice with bone marrow-derived MCs (BMMCs) derived from WT animals. Table 2 summarizes the main tumor development studies that have been conducted in various MC-deficient models. The principal characteristics of MC-deficient animals are also mentioned since the results should be interpreted with caution and the existence of defects on other cell lineages and immune processes in those animals should be taken into account. The studies mentioned in Table 2 indicate that, as in human tumors, MCs can play a positive and/or a negative role in pre-clinical models of tumor growth. 

To explain the dual role of MCs in tumor development, it is necessary to consider the cellular and molecular composition of the tissue where the tumor emerges and develops. This niche is known as the tumor microenvironment (TME), a complex interactive scenario formed by the transformed cells themselves and a highly heterogeneous population of non-tumor cells, which can reside in the tissue or be recruited into the tumor. Among these non-tumor cells are fibroblasts, pericytes, adipocytes, endothelial cells, and certainly immune cells [87]. In addition, the TME includes surrounding blood and lymphatic vessels, extracellular matrix (ECM), signaling molecules such as cytokines and chemokines, and tissular metabolic alterations due to the high proliferation rate of tumor cells, among which are low oxygen concentrations and a decrease in pH [88]. All these components induce a chaotic and hostile environment that not only modifies the phenotype of tumor cells and immune cells but also enhances the aggressiveness of cancer, supporting the idea that tumor development is strongly dependent on cellular interactions within the TME [87,89]. 

In the following sections, we will first discuss the stimuli and signal transduction pathways that have been involved in MCs’ migration to tumors. Then, we will also discuss the available data related to the influence of TME conditions (such as hypoxia) in the phenotype of MCs, the effect and signaling cascades activated by selected tumor-secreted mediators on MCs, the role of MC-derived mediators on tumor angiogenesis, lymphangiogenesis and fibrosis, together with the evidence linking MC activity with metastasis generation. Finally, the therapies that have been proposed to control tumor growth and dissemination by targeting MCs will be discussed.

## 3. Signaling Pathways and Chemotactic Molecules Involved in Mast Cell-Migration to Solid Tumors

Cell migration is a fundamental process that allows the translocation of an individual cell or a group of cells through tissues or fluids. For many years, MCs were considered as tissue-resident cells that, after migrating from the bone marrow (as mast cell progenitors, MCp) to vascularized tissues, finish their differentiation under the influence of locally produced mediators. More recent evidence indicates that MCs can migrate in response to mediators produced in distinct pathological conditions. In cancer, MCs migrate to different types of tumors, including lip squamous cell carcinoma, brain tumors, gastric cancer, melanoma, and glioblastoma [90,91,92,93,94], among others. 

### 3.1. Tumor-Derived Cytokines and Growth Factors That Promote MC Migration to Tumors 

Even though numerous MC chemoattractants have been described [95], only some of them have been studied in the scope of oncological processes. One of the best-characterized MC chemoattractants is SCF, which promotes MC survival and is produced by tumor cells. Human glioblastoma cells, hepatocarcinoma cells (H22), mammary carcinoma cells (AC2M2), and colon cancer cells (HT29 and Caco2) produce SCF to stimulate the infiltration of MCs into the tumors. The molecular mechanism involving MC chemotaxis to SCF involves the activation of the SCF receptor (c-Kit), that, after the activation of its tyrosine kinase domain, activates complex signaling pathways that lead to MC movement. In the signaling cascade of the c-Kit receptor that leads to MC chemotaxis, the activity of Src family kinases, the phosphorylation of adapters, and enzyme recruitment have been reported [94,96,97,98]. Recently, a role of the protein 4.1R on MC chemotaxis towards SCF was found. Utilizing BMMCs obtained from WT and 4.1R-deficient mice, it was observed that chemotaxis towards SCF and spreading on fibronectin were reduced in the absence of the 4.1R protein [99]. Since that polypeptide belongs to a family of hub proteins that couple distinct membrane proteins to the cytoskeleton [100], the formation of protein aggregates in the plasma membrane of MCs when migration to SCF was proposed.

Besides SCF, many chemokines released by solid tumors can also stimulate the migration of MCs. It was previously shown that the pharmacologic inhibition of CXCR4, the receptor of chemokine CXCL2, inhibits MCs’ migration towards pancreatic adenocarcinoma in mice, increasing the survival of affected individuals [36]. In tumor tissue and conditioned media (CM) of pancreatic cell lines, the inhibition of CXCR4 by a neutralizing antibody decreased MC migration towards gastric cancer CM [26]. In both cases, the chemokine CXCL12 was found to be responsible for MCs chemotaxis. Other relevant chemokines secreted by tumors are CCL2, CCL5, CCL11, and CCL15. A study performed by Giannou A.D. and cols. demonstrated that Lewis lung carcinoma cells (LLC) significantly expressed CCL2 and induce MC chemotaxis into pleural cavities [63]. It was reported that in infection induced by human papillomavirus type 16 (HPV-16), the viral oncoprotein E7 induces the secretion of CCL2 and CCL5, contributing to MC recruitment [62]. In addition, CCL5 and CCL11 are highly expressed in Hodgkin leukemia cell lines and uterine cellular leiomyoma cells, inducing the migration of MCs towards CM [101,102]. Similarly, CM of the cancer cell line HT29, which constitutively expresses CCL15, induces MC migration that is significantly diminished in the presence of a CCL15-blocking antibody [98]. All these data suggest that tumors release chemokines capable of recruiting MCs into the TME by activating chemokine receptors.

Interestingly, it has been shown that angiogenic factors synthesized by tumor cells induce MC chemotaxis. For example, in 1995, Gruber et al. reported that murine BMMCs lines (C1.MC/C57.1) were able to migrate in response to VEGF, platelet-derived growth factor AB (PDGF-AB), and basic fibroblast growth factor (bFGF) by a mechanism requiring tyrosine phosphorylation of receptors and downstream effects [103], indicating that factors acting on endothelial cells to alter vascular permeability also recruit MCs. Furthermore, Melillo and cols. demonstrated that MCs migrate towards the CM from thyroid carcinoma cell lines and that blocking VEGF-A with a neutralizing antibody mitigated MC migration, concluding that VEGF-A participates in MCs chemotaxis [48]. About FGF, it was shown that the PTX3-derived small molecule FGF trap significantly decreases the recruitment of MCs to the periphery of tumor lesions of murine models of prostate cancer, suggesting that FGF can induce MC migration [104]. On the other hand, adrenomedullin (AM), a 52-amino acid peptide with vasodilator effects, was increased in renal carcinoma (RC) patients, correlating with an increase in MC density in AM-positive RC tissues compared to AM-negative RC tissues, suggesting that AM acts as MCs’ chemoattractant. Moreover, in vitro chemotaxis experiments confirmed that AM modulates MCs’ recruitment to RC tumors via the phosphatidylinositol-3-kinase (PI3K)/AKT/GSK3β/AM signaling pathway [44]. 

Other chemoattractants increased in solid tumor sites are complement products (C3a, C5a), transforming growth factor β (TGF-β), platelet-derived growth factor (PDGF), adenosine, chemokine CXCL10, or interleukins (IL)-8 and 37, and lipid mediators including sphingosine-1-phosphate (S1P), lysophosphatidylinositol (LPI), prostaglandin E2 (PGE2), or leukotrienes (LTB4) [105,106]. Except for lipid mediators, which will be discussed in the next section, the effects of those molecules as MC migration agents towards malignant tumors have not been elucidated and should be a matter of further studies in the future. 

### 3.2. Bioactive Lipids as Key Molecules Involved in MC–Tumor Interactions

**Figure 2:**
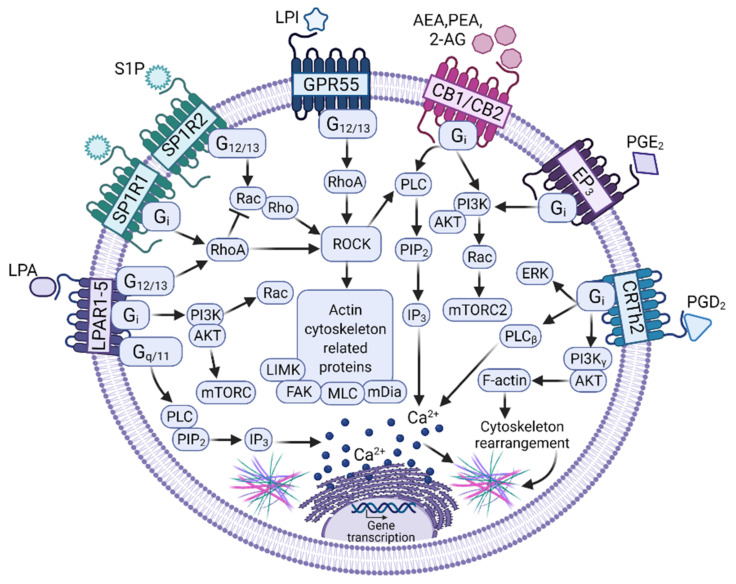
Signaling pathways activated by bioactive lipids to induce migration in MCs. Distinct bioactive lipids secreted by tumor cells induce MCs’ migration towards solid tumors by the activation of signaling pathways leading to actin cytoskeletal re-arrangements. Those pathways are initiated by the binding of the ligands Lysophosphatidic acid (LPA), Sphingosine-1-Phosphate (S1P), Lysophosphatidic acid (LPI), Anandamide (AEA), 2-araquidonoyl-glycerol (2-AG), Phosphatidyl-ethanolamine (PEA), and Prostaglandins E2 or D2 (PGE2 or PGD2) to their respective G protein-coupled receptors (GPCRs), which activate heterotrimeric G proteins (G_i_, G_q/11_, G_12/13_) to initiate canonical signaling cascades mainly controlled by the Rho and Rac family of small GTPases and the phosphatidylinositol 3-kinase (PI3K). In addition, activation of Phospholipase C (PLC) and calcium mobilization is required (see text for details). The modifications to MCs’ transcriptome induced by bioactive ligands and the consequences of their respective GPCRs on MCs’ phenotype remains to be fully analyzed. Figure made using BioRender, agreement number XQ23G72YOM.

Novel evidence indicates that different lipid mediators act as MCs’ chemotactic molecules and could modulate the activity of MCs in pro-inflammatory niches and TME (Figure 2). Those include S1P, lysophosphatidic acid (LPA), PGE2, PGD2, LTB4, LTD4, and LTC4. Bioactive lipids are molecules that trigger different GPCR-mediated signaling pathways and regulate immune and inflammatory responses, as well as cellular homeostasis [107]. According to Chiurchiù and cols. those lipids are classified into four groups: lysophospholipids/sphingolipids, classical eicosanoids, endocannabinoids, and specialized pro-resolving mediators [108]. Regarding MC–tumor communication, only the first three categories have started to be explored.

The group of lysophospholipids/sphingolipids, characterized by containing phospholipids with a single acylated chain and a glycerol or sphingosine backbone, has gained importance in recent years due to the discoveries indicating that they are important regulators of immune cells [109]. Those include S1P, a very well characterized compound that promotes MCs’ migration, and other molecules, such as LPI or LPA that also seem to influence MCs’ migration. 

S1P is a sphingolipid metabolite produced by the sphingosine kinases (SphKs) 1 and 2 that is increased in TME and was considered as a promising therapeutic target in the control of cancer growth [110]. S1P is secreted by tumor cells and was found in high concentrations in TME [111]. From the extracellular space, S1P exerts its effects through the activation of five GPCR receptors named S1P receptors (S1PR) 1–5. MCs express S1PR1, S1PR2, and S1PR4 [112]. S1PR1 is involved in the migration of MCs toward low concentrations of antigen, whereas S1PR2 participates in FcεRI degranulation [113,114]. 

To induce migration, S1PR1 triggering leads to the activation of heterotrimeric G_i_ proteins, which can modulate different pathways leading to cell survival through the PI3K/Akt pathway, cell migration through the PI3K and Rac pathways, and cell proliferation through the ERK1/2 pathway. In contrast, S1PR2 is coupled to Gi, Gq, and G_12/13_ [115]. Loss of S1PR resulted in a decreased chemotactic motility, whereas loss of S1PR2 inhibited MC degranulation. The concentrations of S1P needed for degranulation were higher than those needed for chemotaxis [113] and it was suggested that MCs migrate to target tissues with low S1P concentration gradients in inflammatory conditions. However, after reaching the target, higher S1P concentrations would prevent further migration and the cells could start to degranulate in response to a more extensive ligation of S1PRs [116], a situation of critical relevance to understanding the role of MC physiology inside the TME. 

Besides S1P, LPI has emerged as an important bioactive lipid with a regulatory role in cancer. Increased levels of LPI have been reported in patients with colon, prostate, breast, and ovarian cancer, suggesting that transformed cells can produce LPI [117,118,119,120]. Transformed cells also can respond to LPI. The effect of this mediator on cell migration has been reported mainly in transformed cells, such as breast, prostate, and colon cancer models, where LPI leads to the formation of filopodia and enhances cell polarization and migration, resulting in increased metastasis [118]. To induce migration, LPI interacts with the GPR55 receptor, considered as a putative cannabinoid receptor since it recognizes classical ligands of this system [121]. This receptor is coupled to G_12/13_ proteins and its engagement leads to the activation of GTPases such as RhoA, Cdc42, and Rac1, which regulate actin cytoskeleton remodeling, cell shape, polarity, and migration. The expression of GPCR55 in MCs has already been reported [122,123] and its activation (together with the cannabinoid CB2 receptor) inhibits the FcεRI-mediated degranulation and cytokine expression in this cell type [123]. Any possible involvement of LPI on MC migration to tumors remains as an open question. 

Another lysophospholipid is LPA, which requires two phospholipases for its synthesis: autotaxin (ATX) and specific-LPA phospholipase A1 [124]. Interestingly, the ATX/LPA axis is over-expressed in oncological diseases such as melanoma, breast cancer, renal carcinoma, and neuroblastoma [125,126,127,128]. Furthermore, it was observed that LPA participates in angiogenesis, cell proliferation, growth, survival, immunomodulation, migration, and invasion [129]. The actions of LPA are mediated by six specific GPCRs, namely LPAR 1–6, and two unspecific GPCRs—the GPR87 and P2Y5 receptors. Specific LPA receptors are coupled to the G_s_, G_i/o_, G_q/11_, and/or G_12/13_ proteins [130]. Interestingly, it was observed that MCs express the LPAR1-5 gene [131]. In those cells, LPA induces the expression of proinflammatory chemokines through LPAR2 as well as proliferation through LPAR1 and LPAR3 in human MCs, and the release of histamine in rat peritoneal MCs [132,133]. In addition, MCs express ATX, suggesting they are also capable of synthesizing LPA [134]. Unfortunately, LPA’s effects on MC migration to TME have not been studied; however, evidence suggests LPA could be a MCs chemoattractant and that its participation in MCs’ migration to solid tumors is possible. 

Regarding classical eicosanoids, PGE2 is the most abundant in oncological processes. Expression of PGE2, COX-1, COX-2, or its microsomal PGE synthetase (mPGES-1) is highly increased in human epithelial ovarian cancer cells, squamous cervix carcinoma, adenocarcinoma tissues, papillary thyroid cancer cell lines, and human hepatocarcinoma tissues [135,136,137,138]. PGE2 participates in angiogenesis, cell differentiation and proliferation, immunoregulation, migration, invasion, and metastasis, acting on different cells from the TME [139]. PGE2 binds to GPCRs receptors, namely EP 1-4. In distinct preparations of MCs, the expression of all those receptors has been observed and evidence indicates that the effects of PGE2 on MCs are diverse in inflammatory contexts. For example, in the MC-9 cell line, PGE2, through EP1 and/or EP3 activation, enhances FcεRI degranulation and selected cytokine production [140]. However, when analyzed in the murine OVA-induced allergic asthma, the EP3 receptor was shown to play an inhibitory role in MC activation, since mice that were deficient in that molecule (Ptger3^−/−^), but not those deficient in the other PGE2 receptors, presented an enhanced allergic response accompanied by increased cell recruitment to inflamed lungs and higher levels of histamine and cysteinyl leukotrienes [141]. Since the mentioned mediators are mainly produced by MCs, the authors suggested that PGE2, acting on an EP3 receptor on that cell type, modulates MC-dependent pro-inflammatory mediator release. That hypothesis was confirmed in a model of PGE2-induced swelling, where the effect of that lipid was prevented in MC-deficient mice and recovered when they were reconstituted with BMMCs from WT but not from EP3-deficient animals [142]. When tested on BMMCs or peritoneal MCs, PGE2 promoted histamine release and IL-6 production with the participation of the EP3 receptor coupled to Gi/o, calcium mobilization, and PI3K activation in WT but not in EP3-deficient cells [142]. On the other side, in vivo studies showed that PGE2-induced MC migration is regulated via mTORC2/PI3K/Rac [143]. Again, there are no specific studies addressing the question of whether PGE2 induces MC migration towards malignancies; however, evidence suggests that its participation should be considered. 

Another relevant prostaglandin is PGD2, an important anti-inflammatory mediator. It is produced by the cyclooxygenase (COX) enzyme and by the hematopoietic PGD synthase (H-PGDS) encoded by the *Hpgds* gene. In high-grade serous ovarian cancer, gastric cancer tissues or Lewis lung carcinoma, PGD2 and H-PGD2 synthase are highly expressed [144,145,146]. MCs express PGD2 receptors: the classic DP1 receptor and the DP2 receptor, also known as the chemoattractant receptor-homologous molecule expressed on Th2 cells (CRTh2). The migration of PGD2-stimulated MCs to cancer cell lines has not been demonstrated. However, PGD2 production by MCs is important in anti-inflammatory responses and acts as an antiangiogenic factor in lung carcinoma [146]. Furthermore, the activation of CRTh2 by PGD2 or the specific agonist DK-PGD2 increased calcium mobilization and ERKs kinase phosphorylation in BMMCs, inducing their migration in both in vitro and in vivo experiments [147]. Interestingly, anaphylactic reactions and inflammatory symptoms caused by MC activation via IgE/Ag complexes were higher in *Hpgds^−/−^* mice than WT animals, indicating that MC-derived prostaglandin D2 diminish the intensity of pro-inflammatory reactions [148], and potentially, could exert limiting effects on tumor growth. 

Finally, the group of endocannabinoids (ECs) has gained popularity in recent years and although its role in MC tumor migration has not been elucidated, there is evidence of the expression of classical cannabinoid receptors in MCs [149]. The best-studied ECs are N-arachidonoylethanolamine (AEA) and 2-arachidonoylglycerol (2-AG), although other ECs are also relevant, such as palmitoylethanolamide (PEA). ECs perform their functions by binding to canonical CB1 and CB2 GPCRs. MCs express both CB1 and CB2 receptors. When CB2 is activated, it induces negative regulatory effects on MCs’ activation, while CB1 contributes to the suppression of secretory responses [149]. ECs also limit excessive MC maturation and activation in human skin in situ and regulate SCF expression via CB1 stimulation [150]. In addition, AEA inhibits FcεRI-dependent degranulation and cytokine synthesis through a possible CB2 and GPR55 dimer activation in MCs [123]. On the other hand, 2-AG can inhibit antigen-induced histamine release in guinea pigs’ MCs via CB2 activation, and LPS-induced TNF-α release in BMMCs [151,152]. Regarding migration, there are no studies that demonstrate that 2-AG or AEA stimulate migration of MCs; however, PAE was shown to reduce MC density and degranulation in traumatic brain injuries collected from mice [153]. Whether this effect is generated for an antiproliferative mechanism or an inhibition of MC migration is not clear. Since cannabinoids exert anti-inflammatory effects in distinct pathological conditions, further studies are needed to examine the role of ECs in MC migration and activation in the context of cancer.

## 4. Influence of TME Conditions on MC Physiology: Can We Talk about Mast Cell Polarization? 

**Figure 3:**
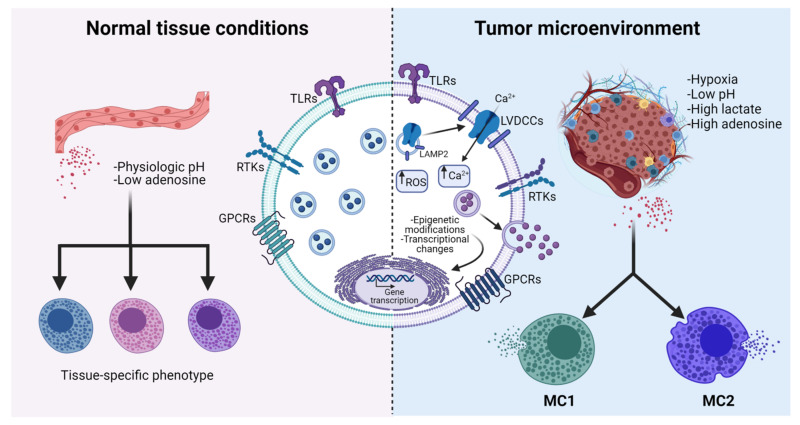
Possible MC polarization towards MC1 and MC2 phenotypes caused by the influence of TME. MCs located in normal organs establish communication with surrounding cells by sensing the production of tissue-specific molecules. This interaction allows the production of a limited number of mediators, which favors tissue homeostasis (**left** panel). Under the influence of extreme conditions that are prevalent in the TME (such as hypoxia, oxidant environments, and high concentrations of adenosine), MCs suffer changes that include the increase in intracellular ROS, the translocation of the L-type voltage-dependent calcium channel (LVDCC) from LAMP2 positive reservoirs to the plasma membrane (see Section 4.1), and possible epigenetic and transcriptional modifications. Current explanations of diverse experimental observations on the role of MCs on tumor growth include the possible differentiation to, at least, two different phenotypes, called MC1 (anti-tumoral) and MC2 (pro-tumoral) ones (**right** panel). See details in the text. Figure was made using BioRender, agreement number MH23G74PX.

Once MCs reach tumors, they become, as in other tissues, sensitive to the influence of locally produced mediators. Transcriptomic profiles have revealed that MCs show high heterogeneity in gene expression across tissues. It has been demonstrated, in both humans and rodents, that depending on the signals released from the tissue where they reside, MCs express surface markers and signaling molecules that make them unique compared with MCs that reside in other anatomical locations [154,155] (Figure 3). This tissue-dependent phenotypic plasticity and transcriptomic heterogeneity cluster MCs in a very distinctive group of immune cells. Due to this plasticity, it was hypothesized that, in pathological conditions such as malignant tumors, MCs could display different phenotypes, depending on the stimuli they receive from the TME [156]. For example, Presta and cols. hypothesized that MCs could adopt two different phenotypes, similar to macrophages. These two hypothetical phenotypes are called MC1 and MC2, related to pro-inflammatory and anti-inflammatory secretory profiles, respectively. According to this novel idea, the MC1 phenotype would be characterized by a low expression of IL-10 and the ability to induce a high inflammatory cell infiltrate, while the MC2 phenotype would be characterized by an elevated synthesis and release of IL-10, leading to a weak inflammatory cell infiltrate [157]. Moreover, the MC2 group of MCs could promote the recruitment of regulatory T cells (Tregs), since some studies have demonstrated that tryptase, a classical marker of MCs, has a positive correlation with the Treg marker FOXP3 in human gastric cancer (GC) and hepatocellular carcinoma [29,158]. Interestingly, it was observed that expression of TGF-β correlates with tryptase-positive cells and that Tregs express the TGF-β receptor in GC biopsies, suggesting that this cytokine could be involved in the crosstalk between MCs and Tregs [158]. Similar to M2 macrophages, the MC2 subset of MCs could foster tumor progression by promoting an immunosuppressive microenvironment that hinders cytotoxic responses of NK and T cells against tumor cells. In accordance with this, Varrichi and cols. suggested that MC2 cells play a pro-tumorigenic role, whereas the MC1 subset is anti-tumorigenic (Figure 3). Interestingly, the authors highlighted that the complex biochemical milieu of the TME could polarize MCs towards these two phenotypes [159]. Extensive research is needed to identify key molecules or environmental conditions that could orchestrate the possible phenotypic changes in MCs that lead to polarization towards the MC1 or MC2 phenotypes.

In the following sections, we address some characteristics of TME that could impact the phenotypic changes of MCs. Due to hypoxia being a distinctive feature of the TME, we will place special emphasis on the effects that this condition may have on the MC phenotype.

### 4.1. Hypoxia and MCs: A Dangerous Connection in Cancer?

Hypoxia is a condition in which oxygen (O_2_) demand exceeds supply in a particular tissue or organ. Maintaining optimal O_2_ levels is a matter of life and death for aerobic organisms; thus, hypoxic cells have to modify their transcriptome to adapt to low O_2_ levels, altering the expression of genes involved in cell survival and apoptosis [160]. The master regulator of the cellular response to hypoxia is the hypoxia-inducible factor 1 (HIF-1), which is a heterodimeric transcription factor consisting of a constitutively expressed β-subunit and an O_2_-regulated α-subunit. Under normoxic conditions, HIF-1α is continuously degraded through the ubiquitin–proteasome pathway, but hypoxic conditions lead to HIF-1α’s stabilization, accumulation, and nucleus translocation, where it forms a heterodimer with HIF-1β. This transcriptional complex binds to hypoxia-response elements (HREs) to induce the expression of several genes related to multiple processes, such as glucose transport, energy metabolism, erythropoiesis, and angiogenesis [161]. 

**Figure 4:**
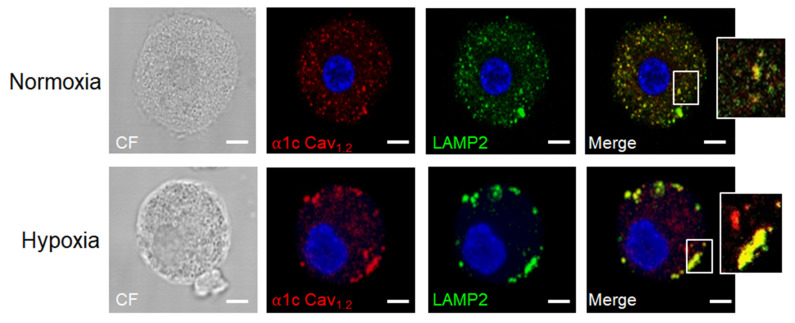
Example of molecular changes occurring in MCs under hypoxic conditions. Cav1.2 subunit of L-type voltage-dependent calcium channels (LVDCC) is associated with LAMP2-containing vesicles and translocates to the plasma membrane. Bone marrow-derived MCs (BMMCs) were exposed to low-oxygen conditions (1% O_2_) for 24 h. After that time, cells were harvested, fixed by standard methods, and the localization of the Cav1.2 subunit of LVDCC and the lysosomal marker LAMP2 was performed utilizing specific antibodies and confocal microscopy. A representative picture of at least five taken with different BMMCs cultures is shown and selected sections were amplified (white rectangles). Photograph was taken by AIS. Scale bar = 2 μm.

It has been well documented that hypoxia is a universal feature of the interior of solid tumors, where O_2_ concentrations can be as low as 1–2% [162,163]. TAMCs can be exposed to this condition and should respond to this important TME feature. Phenotypic changes triggered by hypoxia in this cell type remain poorly explored. A pioneer work demonstrated that hypoxia per se increases IL-6 release but does not induce degranulation in human culture MCs. Importantly, IL-6 was critical for MC survival, since IL-6 neutralization significantly decreased the MC viability under hypoxia [164]. More recently, results in our laboratory demonstrated that BMMCs exposed to 1% O_2_ exhibited an increase in the secretion of the chemokine CCL2 as early as two hours after exposure to hypoxia, suggesting that this condition can modulate the secretory profile of MCs. Moreover, hypoxia was accompanied by an important production of reactive oxygen species (ROS) that was necessary for an increase in intracellular calcium concentration in hypoxic BMMCs. Interestingly, the hypoxia-induced calcium increase was not only dependent on ROS formation, but it was sensitive to nifedipine, a selective inhibitor of L-type voltage-dependent calcium channels (LVDCCs). In the same study, it was observed that a 24-h exposure of BMMCs to 1% O_2_ promotes the translocation of the α1c subunit of the Cav1.2 channel from intracellular pools to the plasma membrane [165] and that this phenomenon occurs through vesicles positive to lysosome-associated membrane protein 2 (LAMP2) (Figure 4). Interestingly, the translocation of LAMP proteins has been associated with the activation of MCs and basophils [166,167], and in this regard, it is possible to hypothesize that hypoxia may promote the mobilization of MC granules towards the plasma membrane and the subsequent release of mediators that could have an important impact on the TME. However, further investigations are required to test this interesting hypothesis. In particular, because tumor mass seems to be subjected to cyclic hypoxia [168], the study of the effect of that particular condition on phenotypic changes in the MC secretome remains as a promising source of information about changes that this immune cell type suffers inside the TME.

## 5. Tumor-Derived Molecules That Activate MCs

Tumors secrete several mediators that contribute to creating specific microenvironmental conditions to promote cell proliferation, angiogenesis, metastasis, and immunosuppression. Signaling pathways involved in the communication between MCs and tumors involved in the secretion of cytokines and chemical mediators by this cell type have not been fully described. In the next section, we summarized some of the main tumor-produced mediators able to activate MCs, emphasizing the signaling pathways that lead to MC-derived cytokine production. 

### 5.1. Tumor-Derived DAMPs That Activate Toll-like and RAGE Receptors in MCs

During tumorigenesis, tumor cells may be damaged because of efficient anti-tumoral activity mediated by cytotoxic immune cells, or cell death mediated by apoptosis or necroptosis may take place due to oxygen deprivation within the TME. Cell damage and death lead to the release of alarmins or damage-associated molecular patterns (DAMPs), which are released both from necrotic cells and in response to anti-cancer therapies. Examples of those molecules are the calcium-binding proteins family (S100s), the high mobility group box 1 protein (HMGB1), adenosine, IL-33, and others, which interact with several pattern recognition receptors (PRRs), such as Toll-like receptors (TLRs) and receptors for advanced glycation products (RAGE) expressed in MCs [7,169,170,171,172].

The main receptors for HMGB1 are TLR-4 and RAGE [173]. In MCs, TLR-4 receptor activation triggers the MyD88-dependent canonical signaling pathway, leading to the nuclear translocation of IKK-dependent NFĸB and promoting the production of TNF-α. Our group previously showed that, after TLR-4 triggering, IKK also phosphorylates SNAP-23, a SNARE protein involved in the secretion of preformed TNF-α [174]. The release of pre-formed TNF-α requires the activation of PKC, the mobilization of VAMP3-positive vesicles [175], and the activation of the metalloproteinase ADAM-17 (TACE) in an ERK1/2-dependent fashion [176]. The effects of TNF-α produced in solid tumors include the activation of the NFĸB-signaling pathway and the induction of an immunosuppressive phenotype on MCs, characterized by the high expression of PD-L1 in those cells [26]. Thus, the secretion of TNF-α, mediated by TLR-4 receptor activation, may exert positive autocrine feedback and promote an immunosuppressive phenotype of MCs into TME. Additionally, in vitro studies show that, in response to TLR-4 ligands, MCs produce a pro-inflammatory cytokine profile characterized by TNF-α, IL-1β, IL-6, and IL-13 [177], which could contribute to increasing the inflammation inside tumors. Finally, although the role of MCs activated by the HMGB1 ligand has not yet been described in detail, HMGB1 was shown to enhance the pro-tumoral activities of M2 macrophages through a mechanism that is dependent on the RAGE receptor [178], and whether this also happens in TAMCs remains as an interesting open question.

### 5.2. Tumor-Derived Mediators That Activate RTKs in MCs 

Tumor cells secrete diverse growth factors that act in autocrine and paracrine ways to support malignant cell proliferation, angiogenesis, and metastasis [179]. Particularly, SCF released by tumor cells can activate the c-KIT receptor expressed on mature MCs, increasing IL-17 expression and promoting an immunosuppressive TME characterized by the secretion of adenosine and the increase in Tregs cell infiltration in tumor samples [97]. The signaling pathways triggered by the SCF/c-Kit axis include phospholipase C γ (PLCγ) activation, an increase in inositol 1,4,5-triphosphate (IP3), and the phosphorylation of mitogen-activated protein kinases (MAPKs), Akt, and transcription factors such as NFĸB, STAT-5, and STAT6 in MCs [180]. Stimulation of the c-Kit receptor also activates the mTORC1 signaling pathway in a PI3K-dependent fashion, which is essential to the production of cytokines and the chemotaxis of human and mouse MCs (see previous sections) [181].

SCF increases several pro-inflammatory mediators, such as IL-6, TNF-α, VEGF, Cox-2, iNOS, and CCL-2 in BMMCs. Moreover, it was shown that SCF potentiates the response activated via the FcεRI receptor, suggesting that c-Kit and FcεRI act in close crosstalk [182]. Interestingly, crosstalk between c-Kit and the Toll/IL-1receptor (TIR) family member IL-33 receptor (IL-33R) has also been proposed. In a recent work, it was demonstrated that IL-33 cross-activates the c-Kit receptor in human and murine MCs through the formation of a complex formed by c-Kit, IL-33R, and the IL-1 receptor accessory protein. The authors found that c-Kit activation is necessary for the correct signaling pathway triggered by IL-33 since imatinib, an inhibitor of tyrosine kinases including c-Kit, diminished IL-33-induced cytokine secretion. Furthermore, in wild-type BMMCs stimulated with IL-33, the formation of the c-Kit/IL-33R complex only occurred upon SCF stimulation. Interestingly, co-stimulation with SCF plus IL-33 induced higher levels of IL-6 compared to those observed in cells stimulated with SCF or IL-33 alone, suggesting a synergistic effect of these molecules in MC activation [183]. This could be relevant in the context of cancer, since tumor-derived IL-33 activates MCs to produce chemotactic molecules that promote tumor-associated macrophages (TAMs)’ infiltration, which, in turn, supports angiogenesis and tumor growth in a mice model of GC. Interestingly, in GC patients, the transcriptional signature of activated MCs with IL-33 and TAMs’ markers correlates with decreased patient survival, suggesting that IL-33 is a potent tumor-derived molecule capable of activating MCs to foster their pro-tumorigenic role in GC [61]. However, considering that many molecules that are capable of activating MCs are present in the TME, it remains to be clarified whether the triggering of other TIR-family members, such as TLRs, also cross-activates RTKs in MCs and contributes to tumor progression.

### 5.3. Tumor-Derived TGF-β Activate MCs 

TGF-β is a growth factor that regulates key aspects of immune responses. In addition, it promotes fibrotic processes and regulates many aspects of tumor immunology. It is produced by malignant cells and by other cell types present in the TME, including Tregs [184]. TGF-β is a potent chemoattractant of BMMCs, peritoneal MCs, and HMC-1 cells, and this factor is considered a key molecule in the migration of MCs towards tumor tissue [185,186]. The signaling pathways that modulate the migration of MCs are not known in detail. However, the activation of MCs with TGF-β favors a polarized morphology with changes in the cytoskeleton that are mediated by the Rho GTPase signaling pathways that characterize cells that are ready to start migration [186,187]. Other proteins activated in the process of migration of MCs induced by TGF-β are Fyn kinase and the protein phosphatase 2A (PP2A), which promote actin depolarization mediated by cofilin activation [188], and the activation of MAP kinases MEK1 and MEK2 [185]. In vitro studies have shown that TGF-β regulates BMMCs’ proliferation, growth, and differentiation [189]. Moreover, TGF-β inhibits the de novo synthesis of the Kit protein in a Smad-dependent manner and prevents degranulation and cytokine production in response to the aggregation of FcεRI, as well as promoting apoptosis in human skin MCs [190]. TGF-β ligands are also potent antagonists of IL-33-induced MC function in allergic and inflammatory disease [191]. In addition, TGF-β induces the upregulation of MC proteases MCP-1, MCP-6, and MCP-7, and it has also been described that TGF-β increases the production of chymase and tryptase, which can function as activators of extracellular matrix metalloproteinases (MMPs) [192,193,194,195]. In turn, the activity of metalloproteinases is fundamental in the process of metastasis and tumor invasion [196].

### 5.4. Potential Role of Tumor-Derived Adenosine and Neuropeptides in MC Activation

It is well known that the TME contains multiple soluble factors that can activate MCs [106]. One of them is adenosine, a nucleoside composed of a molecule of adenine attached to sugar ribose through glycosidic linkage. Adenosine is synthesized from ATP by an enzymatic action that involves the ectonucleotidases CD39 and CD73 [197]. It has been observed that adenosine concentrations in the TME are significantly higher than in normal tissue [198]. This adenosinergic milieu promotes an immunosuppressive environment, which suppresses anti-tumor immunity and supports tumor growth [199]. Adenosine plays autocrine and paracrine roles in both tumor and non-tumor cells [170]. In particular, the effects that adenosine exerts on MCs have been addressed using distinct in vitro MC preparations and several models of inflammation, asthma, and cancer (reviewed in [200,201]) These functions are mediated by four GPCRs, named A1, A2a, A2b, and A3. From those molecules, the A3 receptor seems to mediate the most important actions of adenosine on MCs [201]. For example, a direct activation of MCs upon contact with cancer cells, by a mechanism involving an autocrine loop of adenosine, and the consequent signaling pathways triggered by the A3 receptor, including PI3K, Akt, and ERK1/2 MAPK phosphorylation, were shown [202]. Moreover, in a more recent study, it was demonstrated that MCs are activated by cancer extracellular vesicles (EVs) in a CD73- and adenosine-dependent manner, promoting the upregulation of genes involved in tissue remodeling and angiogenesis, which was found to be related to lung cancer progression [203]. Taken together, these data suggest that adenosine plays an important role in the MC activation in cancer, which seems not to be restricted to the presence of adenosine in the TME where MCs reside, but rather, also appears to be involved in the direct contact with tumor cells and in the tumor-derived EVs’ effects on MCs. 

Other soluble factors present in the TME are neuropeptides since distinct studies indicate that peripheral nerves display a major role in the first steps of tumor formation [204,205]. Neuropeptides act as potent cellular growth factors for many cell types, including cancer cells. They are a diverse group of messengers that act as neurotransmitters, paracrine regulators, or systemic hormones. Those include angiotensin, substance P, and vasopressin, among others. Neuropeptides have been involved in the autocrine/paracrine stimulation of tumor cell proliferation and migration, and their expression and synthesis have been demonstrated in some types of cancer, including those of the lung, pancreas, and colon (reviewed in [206]).

Given that nerve terminals are increasingly recognized as important structures for tumor development, and neurotransmitters and neuropeptides are present in the TME [207,208], the latter could activate tumor-resident MCs and control their activation. For example, it is known that MCs can be activated by substance P to induce inflammation in distinct pathophysiological contexts [209] and this can be the case inside malignant tumors. Future research will shed light on this unexplored and relevant aspect of nerve–MCs communication MCs in cancer.

## 6. MC-Derived Mediators That Promote the Recruitment of Other Immune Cells to the TME 

Once infiltrated into the tumors, MC-derived mediators can stimulate the migration of other immune cells to the TME. Among those cell types are T lymphocytes, NK cells, macrophages, DCs, neutrophils, and myeloid-derived suppressor cells (MDSCs) [105]. The infiltration of immune cells in the TME is a key factor in cancer prognosis, due to these recruited cells having immunomodulatory effects that can promote or impair tumor growth. For example, the presence of MCs was associated with higher Treg cells infiltration in a model of hepatocarcinoma, suggesting that MC-derived mediators can induce the migration of Tregs cells towards tumors [97]. In addition, in a colon cancer model, activated IgE/Ag BMMCs induced the migration of MDSCs and this was partially mediated by leukotrienes since their inhibition with Montelukast (a leukotriene receptor antagonist) reduced the migration of MDSCs [210]. Additionally, MCs promote the infiltration of IL-17-producing MDSCs, which, in turn, recruit Treg cells and foster immunosuppression in the TME [211]. Importantly, intratumoral tryptase-positive-MCs have been shown to be the main source of IL-17 in some solid tumors [25,30]. In line with these observations, samples from patients with colorectal cancer showed a negative correlation between CD8+ T cell infiltration and MCs’ infiltration, suggesting that the lower the number of MCs in the tumor, the greater the anti-tumor response and better the prognosis of colorectal cancer [212]. Finally, attracted by MC activation, macrophages—the phagocytic cells par excellence—infiltrate into the TME. IL-33-activated-MCs or PI3K/AKT signaling pathway-activation in MCs induce the production and secretion of chemoattracting molecules, stimulating the activation of macrophages as well as promoting their recruitment and the production of macrophage-attracting factors such as Csf2, CCL3, and IL-6, thereby inducing gastric and colon cancer cells’ invasion [61,213]. All these studies suggest a pro-tumoral role of MCs based on inducing the recruitment of immunosuppressive cells that lead to poor defense against tumors. 

On the other hand, MC-derived mediators that induce the recruitment of immune cells that are able to fight against tumors have also been reported. In melanoma tumors, MC-derived CCL3 induced the recruitment of CD8+ T cells and NK cells [53]. Moreover, the secretion of CCL2, which is dependent on the activation of the TLR-7 receptor in MCs, promotes the recruitment of plasmacytoid dendritic cells (pDCs) to the tumor niche where they differentiate to tumor-killing effector cells [214]. Finally, a recent study demonstrated that LPS-activated melanoma-resident MCs can secrete CXCL10, which, in turn, recruits tumor-infiltrating effector T cells (TILs) and initiates melanoma immune defense [55].

Summarizing, MCs are important recruiters of TME-related immune cells. It should be considered that, in addition to the characterized mediators, others could also induce migration of these cell types and that they should also be studied in the context of neoplastic processes, both to know their role in cell trafficking and to understand their effects on those cell types within the tumor, which will help to elucidate the role of MCs in cancer.

## 7. MC-Derived Mediators That Participate in Angiogenesis: The Case of the Production of VEGF 

Hypoxia and other conditions existing in the TME, together with tumor-derived mediators, lead to the production of pro-angiogenic molecules. From those, VEGFA is one of the most important, since it activates the VEGF receptor 2 (VEGFR2) located in endothelial cells (ECs) from blood capillaries and leads to changes in their phenotype, causing the differentiation of tip cells that move across the extracellular matrix and generate new vessel sprouts. It has been shown that malignant (but not benign) tumors induce strong angiogenic processes through a mechanism known as the “angiogenic switch” that includes the formation of a dense network of blood vessels inside the tumors. However, anti-angiogenic molecules are also produced, modulating the process of new vessel formation inside the tumor mass (for a review, see [215]).

From initial observations of tumor mass composition, it was proposed that resident immune cells could provide tumor cells with pro-angiogenic factors [216]. Although the participation of MCs in tumor growth and angiogenesis has been accepted due to the fact this cell type can secrete distinct molecules that lead to the formation of new blood vessels, the mechanism by which MCs synthesize and secrete VEGFA and other pro-angiogenic factors that contribute to tumor angiogenesis remains poorly described. MCs are attracted to sites where angiogenesis is produced, as was previously shown with the angiogenesis that occurs on the chorioallantoic membrane model on eggs [217]. In that model, the addition of VEGF (then known as the vascular growth factor) increased the number of MCs forty times 24 h after the initial administration of VEGF, although no direct participation of MCs on angiogenesis was observed. On the other hand, MCs have been shown to produce VEGF. For example, the human mast cell line HMC-1 stimulated for 24 h with PMA/A23187 produced mRNAs for VEGF121, VEGF165, VEGF189, and VEGF206. Moreover, the constitutive presence of VEGF on granules of human skin MCs has also been shown [218]. In line with those studies, it was demonstrated that C1MC/C57.1 MCs expressed the mRNA for VEGF and this increased after triggering of the high affinity IgE receptor (FcεRI) or with the treatment with PMA. Additionally, the presence of VEGF was detected by in situ hybridization of freshly isolated peritoneal mouse MCs (PMCs). Remarkably, VEGF was detected on the supernatants of BMMCs after being treated with monomeric IgE (mIgE) for 4 days and stimulated with a specific antigen [219]. In those conditions, it was demonstrated that VEGF secretion in response to FcεRI crosslinking is a rapid event that can be observed at short times (3 h) after stimulation [219]. In the absence of antigen, monomeric IgE (mIgE) promotes VEGF secretion in BMMCs through a mechanism that requires the activation of Fyn kinase and the translational regulator 4E-BP1, which favors the IRES-dependent translation of VEGF mRNA [54]. The positive effect of mIgE on MC-derived VEGF production was observable in vivo, utilizing the B16-F1 murine melanoma model since the number of peritumoral MCs and intratumoral blood vessel density increased in the presence of mIgE in WT but not in mast cell-deficient c-kit W^sh^/W^sh^ mice (See Table 2). The effects of mIgE on MC-induced tumor angiogenesis were diminished with the anti-VEGF antibody bevacizumab [54]. Signaling pathways activated by mIgE in MCs include the activation of MAPK ERK1/2, JNK, and p38. In addition, mIgE importantly activates Akt (Thr 308 and Ser 473) phosphorylation, the leukotriene synthesis and accumulation of TNF-α, IL-6, IL-4, and IL-13 mRNAs, and the secretion of IL-6. Remarkably, mIgE prevented apoptosis of BMMCs by stabilizing the levels of Bcl-X2 protein [220] and induced the production of ROS [221]. Other stimuli that induce survival of MCs also promote VEGF synthesis in that cell type. For example, IL-6 was found to induce VEGF secretion in human in situ-maturated skin MCs and potentiates FcεRI-induced secretion of that factor [222]. In the same line, IL-33, which promotes MC survival, leads to the production of VEGF in human lung MCs [223,224]. Other mediators produced in the TME also induce VEGF production in MCs. For example, PGE2 induces the synthesis of VEGFA via the EP2 receptor in human cord blood-derived human MCs (CBMCs) [225]. Finally, it is important to mention that recent reports suggest that VEGF and other tumor-produced factors (such as angiopoietins 1 and 2) are associated with MC proliferation since those mediators were found to increase in serum from patients with mastocytosis [226]. The mentioned evidence suggests that intratumoral VEGF could promote MCs’ survival to sustain angiogenesis and, more importantly, that factors that lead to MCs’ survival maintain the production of VEGF necessary for malignant tumor growth. 

Damage-inducing stimuli (such as low dose irradiation, IR) also lead to VEGF synthesis and promote the formation of new blood vessels [227]. Utilizing a limb ischemia model, irradiation with 2-Gy from a cesium source was shown to improve vascular regeneration in a MC-dependent manner, since it was not observed in MC-deficient mice. In addition, irradiation promoted the migration of MC progenitors from the bone marrow to the ischemic site. Remarkably, irradiation-induced effects, such as the recruitment of progenitors, vasculogenesis, and VEGF synthesis in MCs were not observed in MMP-9-deficient mice, showing that the metalloproteinase is not only a potent chemoattractant for MCs, but is also a potent inductor of VEGF synthesis in that cell type. It was suggested that IR induce the expression of selected genes in MCs and stroma cells, leading to the secretion of VEGF and SCF that, in turn, recruit distinct cells (bone marrow precursors, pericytes, MCs, and others) that contribute to angiogenesis [227]. 

Detailed molecular mechanisms leading to MC-derived VEGF production in the high oxidant, immunosuppressive, and hypoxic conditions prevalent in the TME are not fully described. In a recent study, utilizing incubation under a nitrogen atmosphere to induce hypoxia, it was observed that BMMCs accumulate VEGF mRNA [165]. In addition, using cobalt chloride (CoCl_2_) to induce chemical hypoxia, it was shown that BMMCs synthesize and secrete significant amounts of VEGF. Interestingly, VEGF production required the activation of Src family kinases and the generation of ROS, since it was diminished in Fyn-deficient BMMCs and prevented by several antioxidants [228]. Altogether, those data suggest that signaling pathways leading to VEGF production in MCs under hypoxic conditions include the generation of ROS and activation of Src-family kinases (at least Fyn). Since it was reported that mIgE also induces the production of ROS and the activation of Fyn kinase [220], it is possible to speculate that common pathways could be activated by hypoxia and mIgE and may contribute to VEGF secretion and MC survival. Future research will give light to the effect of low O_2_ concentrations on MC survival and the role of mIgE on the production of pro-angiogenic factors in the TME.

## 8. MC-Derived Mediators and Signaling Pathways That Regulate Lymphangiogenesis 

**Figure 5:**
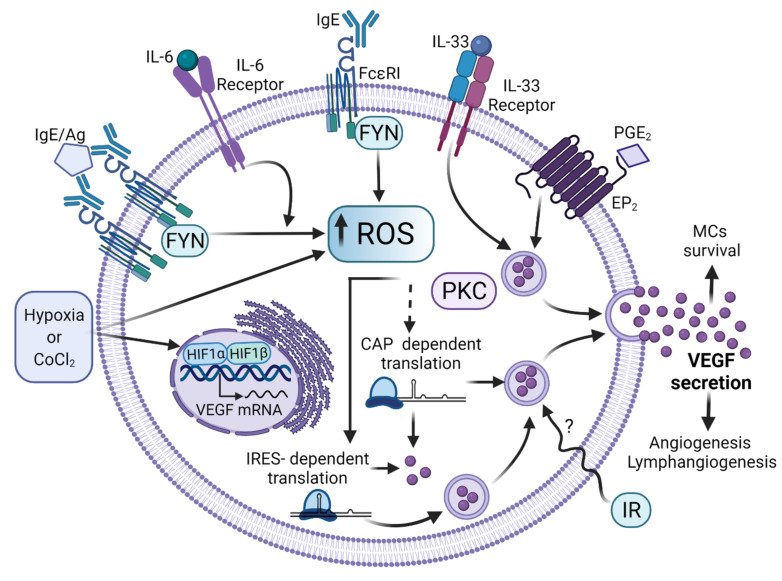
Signaling pathways leading to VEGF synthesis and secretion in MCs. After distinct stimuli (some of them found in TME), MCs secrete VEGF to promote the formation of new blood vessels. Diverse ligands produced in TME or other conditions have been found to induce VEGF synthesis in MCs by controlling several steps on its synthesis and release. The binding of monomeric IgE to FcεRI and the antigen-dependent crosslinking of that receptor lead to ROS generation through the activation of Fyn tyrosine kinase, which promotes the accumulation of VEGF transcript and its translation through the internal ribosome-binding site (IRES) of VEGF mRNA. IL-33 and IL-6 receptors also lead to VEGF production in MCs, together with the triggering of the PGE2 receptor. Low-level ionizing radiation also leads to VEGF synthesis in MCs and this phenomenon leads to the restoring of blood vessels in damaged tissue. TME conditions, such as hypoxia or its mimicking agent cobalt chloride (CoCl_2_), lead to HIF-1α stabilization and promote VEGF transcription. Other intracellular pathways involved in VEGF synthesis in MCs require increased intracellular calcium levels and the activation of protein kinase C (PKC). Figure made in BioRender, agreement number FT23G75A9J.

The lymphatic vessels are important players in tumor growth and metastasis [229]. As in other tissues, inside the tumors, lymphoid endothelial cells (LECs) must proliferate and migrate to form new vessels under the influence of specific growth factors and chemoattractants [230]. Several mediators have been identified as key players in that process, and most of them are produced by MCs. For example, VEGF-C and VEGF-D, acting through their receptor (VEGFR3 and its co-receptor neuropilin2, NRP2), activate LECs and cause proliferation and migration [231]. Utilizing human lung MCs (HLMCs) obtained from the lung parenchyma of surgery patients, and the human immortalized MC lines LAD-2 and HMC-1, it was found that those MCs produce active VEGF-A, VEGF-C, and VEGF-D. Interestingly, prostaglandin E2 (PGE2), a bioactive lipid that is produced in pro-inflammatory conditions, increased VEGF-C but not VEGF-D in LAD-2 cells. In the same study, it was found that NECA, a metabolically stable adenosine analog, induced the production of VEGF-A, VEGF-C, and VEGF-D in a dose–response manner in the HMC-1 cell line [232]. Those data strongly suggest that, in response to two mediators produced in the TME, MCs produce pro-lymphangiogenic factors that could lead to LECs’ migration and proliferation to form new lymphoid vessels. In the same study, it was found that VEGFR-1 and VEGFR-2 are expressed and active on HLMCs since all members of the VEGF family growth factors and PIGF provoked MC chemotaxis [232]. Main signaling pathways leading to the production of mediators participating in angiogenesis and lymphangiogenesis in MCs are depicted in Figure 5. 

## 9. MC-Derived Mediators That Contribute to the Fibrotic Process 

Fibrosis is a key hallmark of chronic inflammation and the link between inflammation and fibrosis has been extensively studied. This condition is characterized by an excessive accumulation of fibrous connective tissue in an organ. Fibrosis is also a well-recognized characteristic of solid tumors since deposition of extracellular matrix (ECM) stiffens tissue stroma and promotes cell transformation, tumor growth, malignant cell survival, metastasis, and mesenchymal transition (reviewed in [233,234]). ECM deposition also promotes hypoxia and the generation of an immunosuppressive TME and tumor metastasis [235]. In solid tumors, fibroblasts are a major component of TME since those cells are transformed into cancer-associated fibroblasts (CAFs) and secrete several ECM proteins and soluble factors, affecting cancer development (reviewed in [236]).

In the context of cancer, evidence indicates that there is a close interaction of MCs and fibrotic sites of tumors. For example, analyzing biopsies of human breast, head and neck, lung, ovarian, and non-Hodgkin’s lymphoma, it was found that degranulated MCs are located in the fibrous tissue of every tumor [237]. On the other hand, it was found that MC-derived heparin inhibits the proliferation of tumor cells by a mechanism that involves peritumoral fibroblasts [237]. In addition, evidence was obtained indicating that CAFs can recruit MCs to the TME via the CXCL12/CXCR4 pathway in prostate cancer [238]. Once recruited, MCs can enhance some fibroblast functions, such as contracting collagen networks through SCF/c-Kit and increasing collagenolytic enzyme production through IL-1α and TNF-α signaling pathways [239,240].

Inside tumors, MCs can reshape the TME by regulating both cell behavior and changing the ECM organization. Besides heparin, MCs may secrete diverse compounds to foster tumor fibrosis, such as TGF-β, histamine, and tryptase, that activate fibroblasts to produce collagen in fibrosis [159]. TGF-β possesses important pro-fibrotic functions since this cytokine promotes fibroblast migration and proliferation, as well as collagen formation and fibroblast differentiation into CAF. Remarkably, besides its pro-fibrotic effects that can be associated with tumor progression, TGF-β also exerts tumor-suppressive effects by inhibiting cell proliferation and immortalization, as well as inducing cell apoptosis. These protective and cytostatic effects of TGF-β are often lost as tumors develop and progress and this is why the control of TGF-β signaling has been proposed as a therapeutic strategy for cancer therapy (reviewed in [241]). Another pro-fibrotic mediator that is produced by MCs is IL-13, which seems to promote fibrosis via TGF-β-dependent and independent mechanisms. IL-13 can induce TGF-β production and activation in vivo and directly promote fibrosis by stimulating proliferation or collagen production by fibroblasts, as well as CAF differentiation.

TGF-β- and IL-13-positive MCs were observed in human biopsies of the nodular sclerosis subtype of classical Hodgkin lymphoma (CHL). In that study, only the expression of IL-13 in Hodgkin and Reed–Sternberg cells (HRS) was associated with a higher rate of fibrosis and the number of MCs was significantly higher in the group with IL-13-positive (HRS) cells of tumors. A significantly positive correlation was observed between the rate of fibrosis and the number of MCs. The authors hypothesized that IL-13 production by HRS cells may lead to fibrosis and promote MCs’ infiltration and proliferation. This, in turn, might induce the production of IL-13 and TGF-β (probably by MCs), resulting in the observed fibrosis of the nodular sclerosis subtype of CHL [242].

Regarding CCL2 (which is also produced by MCs), it has been shown that it attracts fibrocytes to distinct injuries and, interestingly, the interplay among TGF-β, IL-13, and CCL2 has been observed in the context of pulmonary fibrosis, suggesting that may play a role also in tumor fibrosis [243]. Finally, MC-produced proteases have effects on both the connective tissue cells involved in fibrosis, as well as in the surrounding ECM. These proteases stimulate the proliferation of smooth muscle cells, epithelium, and fibroblasts, as well as promoting fibroblast chemotaxis and myofibroblasts differentiation [244]. Tryptase-mediated stimulation of fibroblast proliferation and ECM protein synthesis (collagen and fibronectin) occurs via the activation of the protease-activated receptor PAR-2 [245]. Moreover, tryptase and chymase can also activate MMP to increase ECM turnover [246,247]. Regarding other proteases, MC-derived MMP-2 and MMP-9 are implicated in the ECM’s degradation and tumor invasion, as well as in the cleavage of some angiogenic factors stored in the ECM, allowing them to be released into the TME [238].

## 10. MC-Derived Mediators as Promoters of Metastatic Processes 

To explain how tumor cells can migrate from a primary site to colonize specific distant sites to grow and form metastases, it was proposed that organs that will be metastatic niches are previously modified by factors secreted by primary tumors, where vascular permeability and angiogenesis importantly participate in this process [248]. In 1889, Stephen Paget postulated the theory of seed (cancer cells) and soil (host microenvironment) to explain that metastasis depends on the interaction of cancer cells and the metastatic niche [249]. More recent information indicates that tumors transform the microenvironment of distant organs, which in turn undergo adaptation with the subsequent secretion of molecules that promote the growth of tumor cells before they reach the metastatic niche [250,251]. 

Several epidemiological studies have associated the presence of MCs with the acquisition of aggressive phenotypes in different types of cancers including gastric, pancreatic, and colorectal, while a controversial role has been suggested in breast, lung, and prostate cancers. It was proposed that TME is a complex system where the interaction of different cell types including infiltrating immune cells such as MCs are essential for tumor growth, invasion, and metastasis, determining tumor fate [252]. 

It was shown through a meta-analysis that a high intra-tumor infiltration with tryptase-positive MCs was associated with a decrease in overall survival in patients with hepatocarcinoma and lung cancer, while an association that was not statistically significant was observed for colorectal, esophageal, and prostate cancer, as well as melanoma. Moreover, a significant association of a high MC density was associated with shortened disease-free survival of colorectal cancer and hepatocarcinoma. When clinical pathological features were assessed, a correlation of high tryptase-positive MC density with lymph node metastasis was found in solid tumors such as lung, liver, and colorectal cancer. These findings reveal that tryptase-positive MC density may play an important role as a prognostic value biomarker related to patients with worse clinical outcomes [253].

In a study where MC density was assessed and associated with clinical features and different molecular types of 219 breast cancer samples, it was found that MC infiltration is higher in invasive lobular carcinoma compared with invasive ductal carcinoma [254]. Moreover, breast cancer biopsies that are positive for estrogen (ER) and progesterone (PR) receptors biomarkers harbor a significantly higher MC infiltration than those that are negative for such receptors; however, no differences were observed when comparing HER2-positive and HER2-negative cancer samples. Breast cancer molecular types are differentially associated with the presence of MCs, being luminal A and luminal B subtypes of breast cancer, those with a higher infiltration of MCs when compared with triple-negative breast cancer (ER, PR, and HER2 negative). This is an interesting finding since such molecular types of breast cancer exhibit differences in the immunohistochemical profile that allow their classification and predict patient survival [255]. Further, an enrichment of MCs’ infiltration was observed in breast tumors after the patients were treated with chemotherapy. It was demonstrated that MC density is increased in certain types of invasive breast cancer, which may suggest an early role in metastasis. Nevertheless, in this study, no association was found between tumor growth and lymph node metastasis with MCs’ infiltration [254]. 

Interesting results have been obtained in studies using MC-deficient transgenic mice (c-kit W^sh^/W^sh^), which were crossed with mammary tumor model mice MMTV-Polyoma Middle T antigen (PyMT) (see Table 2). It was observed that lung metastasis was reduced in MC-lacking mice. When MC density was evaluated, MCs were located at the edge of the tumors but not inside of tumor mass [59]. Moreover, another study confirmed that the absence of MCs was associated with an evident reduction in the metastasis dissemination of mammary tumors. Interestingly, the authors argued that the reduction in the metastasis of cancer cells could be explained by a delay in tumor onset in mice lacking MCs compared to those mice with MCs, and not by an intrinsic feature of cancerous cells [60]. Taken together, these results support the idea that MCs play an important role in the metastasis of breast tumors. However, considerations about c-kit W^sh^/W^sh^ mice should be taken into account, since other alterations have been found in these mouse strains (see Table 2).

Previously, it was reported that gastric cancer samples exhibited a high proportion of MCs in comparison to normal gastric tissue. Interestingly, MCs’ infiltration increases with tumor depth, exhibiting the highest proportions in those tumors that invade adjacent structures compared to those that invades lamina propria or submucosal. Subsequently, it was found that MCs are highly represented in tumors with lymphatic and vessel invasion and lymph nodes metastasis [256]. Concordantly, another study showed that the density of MCs that are positive to tryptase was increased in primary gastric cancer tissue and lymph node metastasis compared to normal tissues and lymph nodes; moreover, a positive correlation was found with an increment between microvascular density and MC density [24].

Cervical tumors have been reported to harbor high infiltration MCs that are positive to tryptase in invasive cancer in comparison to normal tissue and cervical intraepithelial neoplasia from grades 1 to 3 [257]. In contrast, Diaconu and cols. demonstrated that neither cervical cancer nor normal cervical epithelium contained MC infiltration, although MCs that were positive to chymase and tryptase were present in the distant subepithelial stroma and peritumoral stroma, which is characterized as a zone of reactive inflammation. Meanwhile, MCs were also present in the upper subepithelial stroma of controls. Further, the authors showed in in vitro studies that the culturing of SiHa cervical cancer cells with a preparation of chymase-tryptase promoted cell detachment. Further, when SiHa cells were grown on fibronectin, it was demonstrated that the effect of the chymase-tryptase preparation was due to the degradation of proteins present in the extracellular matrix (ECM), such as fibronectin. SiHa cell detachment was prevented with SBTI, an inhibitor of chymase. Interestingly, those detached cells were viable since they continued with their normal growth when reseeded in a medium without chymase [258]. In contrast, when keratinocytes were treated with chymase, they became apoptotic [258]. These results suggest that chymase and tryptase secreted by MCs in the peritumoral region may favor the remodeling of the ECM of cancer cells, eventually promoting cancer cell migration, invasion, and metastasis. Therefore, MCs may play a role in advanced stages, contributing to tumor vascularization and the generation of a microenvironment required for cancer cell dissemination and metastasis.

Other in vitro studies have suggested that MC-derived mediators promote metastasis by enhancing cellular detachment from the ECM. H520 and A549 cell lines derived from human lung squamous carcinoma and alveolar basal epithelial adenocarcinoma, respectively, were treated with MC chymase (MCC) in increasing concentrations (0–50 mU/mL). It was observed that MCC reduced A549 cancer cell line viability while H520 cells exhibited a small decrement in that process. Interestingly, when A549 cells were grown in suspension with MCC, cellular adhesion decreased. Then, after several washes, MCC was removed from detached A549 cells, allowing those cells to recover their adhesion capacity. Additionally, a reduction in E-cadherin protein levels, correlating with an increase in MMP-9 levels and activity, was observed in A549 cells treated with 25 and 50 mU/mL of MCC [259]. 

Brain metastasis surges from many types of cancer and commonly from lung cancer, breast cancer, and melanoma and presents low treatment response and poor survival outcomes since it is a cause of progressive neurological disability. The biology of brain metastasis must be deeply understood to improve alternative clinical management. The surrounding microenvironment has been recognized as a factor that significantly influences the behavior of brain metastasis [260]. In brain metastasis, infiltrating immune cells are detected; in particular, it has been shown that the brain metastasis contains an active inflammatory microenvironment, although there is controversial information on its role in promoting the growth and survival of metastatic cells or a favorable prognosis for the survival of the patient. 

It has been shown that MCs have a strong biological impact on the TME. Although constituting a minor proportion of immune cells, upon activation, they release mediators of the immune response. Roy and cols. detected infiltration of MCs in brain metastasis from different primary tumors [261]. Through tryptase staining, the authors demonstrated MCs’ infiltration in almost all the brain metastasis tissues analyzed, although the number of MCs was significantly higher in brain metastasis tissues whose primary cancer origin was lung, breast, and kidney. Moreover, a strong biological impact was demonstrated when brain metastasis cell lines and MCs were cocultured, since brain metastasis recruited MCs, while MCs promoted the growth of brain metastasis cells as well as the acquisition of stem cell characteristics [261]. Interestingly, in this model, migrating MCs became activated with the subsequent degranulation, as demonstrated through the increase in β-hexosaminidase release, as compared with the unstimulated MCs and the overexpression of the MC proteases tryptase (TPSAB1), chymase (CMA1), and carboxypeptidase A3 (CPA3). MC–brain metastasis cell cocultures also augmented the expression of various cytokines in MCs, including IL-8, IL-10, VEGF, and MMP2. It was then proposed that brain metastasis cells secreted chemo-attractants that stimulated MC migration and activation. Furthermore, there was also observed a clear effect of activated MCs in terms of promoting the proliferation, migration, and self-renewal of the brain metastasis cells.

One mechanism by which tumor cells can modify the metastatic niche is through exosomes, which are 30 to 150 nm membrane vesicles released by various cells, including cancer cells and MCs [262]. Exosomes can transfer their content of RNA, proteins, or lipids to other cells, causing an effect on them [263,264]. It has been shown that cancer cell-derived exosomes from melanoma [265] and pancreatic cancer [266] can establish a crosstalk with immune cells. Xiao and cols. demonstrated that exosomes from lung adenocarcinoma cells are taken by MCs that produce the activation of MCs, probably via SCF-c-Kit signaling with subsequent degranulation. Supernatants of MCs treated with lung cancer exosomes showed an increase in the levels of MMP-9, tryptase, IL-6, and TNF-α, which also promoted proliferation and migration of endothelial HUVEC cells. The authors propose, as a mechanism of these effects, that lung cancer exosomes could contain SCF and, when taken by MCs, promote their degranulation via SCF-c-Kit signaling; furthermore, the liberated tryptase, among other factors, could promote endothelial cells’ proliferation and migration [267]. 

Taken together, epidemiological studies demonstrate a correlation of MC density with tumor aggressiveness and poor prognosis. Results suggest that MCs and their derived mediators are determining factors in the evolution of tumor growth, impacting cancer cell spreading and also supporting the maintenance of the metastatic niche.

## 11. Targeting Tumor-Associated MCs to Design Novel Cancer Therapies 

Several studies have suggested the potential use of blocking MCs functions within tumors to cause a reduction in tumor growth and size, and in consequence, improve overall survival. Some compounds that prevent MC activities, either directly or indirectly, are discussed below.

In pancreatic cancer, it was demonstrated that masitinib, a potent tyrosine kinase inhibitor that selectively targets the c-Kit receptor that inhibits MC survival, decreases the cell proliferation of MIA PaCa-2 and PANC-1 pancreatic cell lines, but only when combined with gemcitabine (a nucleoside analog) as a standard systemic treatment of this type of cancer [268]. Moreover, when evaluating tumors formed from MIA PaCa-2 and PANC-1 cells xenotransplanted in mice treated with masitinib and gemcitabine, a reduction in tumor growth was observed, evidencing that both drugs in combination may exert better anti-tumor activities [269]. Preclinical studies involving patients with pancreatic cancer reveal that a combination of masitinib with gemcitabine improves overall survival when compared to patients treated with gemcitabine alone [270]. Moreover, a phase-III randomized trial demonstrated that patients with advanced pancreatic ductal adenocarcinoma had improved overall survival when treated with gemcitabine and masitinib compared to those patients treated with placebo and gemcitabine [271]. This evidence indicates that the use of a c-Kit inhibitor may favor the clinical outcome of pancreatic cancer where MCs may be involved, although further studies are needed to elucidate the specific effect of such treatment on MCs and other immune cells that express c-Kit.

The potential implication in cancer cell migration and dissemination has been attributed to MC-derived tryptase in different types of tumors [105]. It was demonstrated that MC tryptase (MCT) is increased in serum of patients with pancreatic cancers; concordantly, such tumors contain high levels of MCT in comparison with pericarcinomatous tissues, which correlates with a higher microvascular density. In addition, the expression of pro-angiogenic factors such as VEGF, PDGF, ANGPT1, and TIE2 was augmented in tumor tissues. Interestingly, MCT induced the proliferation and vascularization of HUVEC cells through the activation of the ANGPT1/TIE2 pathway. Using mice models harboring tumors of PANC-1 pancreatic cancer cells, a reduction in tumor growth was observed when mice were treated with MCT plus nafamostat (tryptase inhibitor), in contrast to mice treated with only MCT. Moreover, low vessel numbers were appreciated in tumors when mice were treated with MCT and nafamostat, indicating a prominent role of MCT in angiogenesis [272]. This information exhibits valuable therapeutic approaches to control tumor dissemination, at least in pancreatic cancer.

Little is known regarding chymase inhibitors in cancer, although several inhibitors have been suggested for other pathologies [273]. It was previously described that MC chymase (MCC) stimulates cell proliferation and dissemination in many types of cancer including lung cancer [259]. Recently, an in vitro study demonstrated that chymostatin, an inhibitor of chymase, inhibits cell proliferation and exhibits pro-apoptotic effects when evaluated on human H441 lung cancer cells [274]. However, the precise mechanism remains unclear.

It was described that cromolyn sodium is the most characterized MC stabilizer since it prevents MC release of mediators such as histamine [275]. Preclinical studies carried out in mice models of gastric cancer reveal that MC density increases in the submucosal region of antral tumors. Conversely, in mice deficient in MCs, a reduction in tumor growth and cell proliferation, as well as the induction of apoptosis, was observed. Interestingly, a significant reduction in tumor growth, cell proliferation, and angiogenesis was observed when mice were treated with cromolyn compared to those treated with vehicle [61]. These data suggest that MC-released mediators are required for tumor maintenance and establishment, supporting the use of cromolyn as a promising therapeutic agent for gastric cancer. Taken together, the accumulated evidence suggests that the targeting of MCs and their mediators constitutes a source of promising novel therapeutics against cancer. 

## 12. Tumor-Associated MCs: Soldiers on the Front Lines or Instigators of the Flames of Cancer? 

In this paper, we reviewed the state of the art about the complex crosstalk between MCs and tumors. Studies analyzing their presence in tumor biopsies and investigations using MC-deficient animal models have made clear that MCs contribute, in many aspects, to tumor biology. Research on the mechanisms of MC recruitment to tumor mass, TME-induced changes in MC phenotypes, and autocrine loops of MC-synthesized molecules are complemented with in vitro studies analyzing the particular effect of tumor-derived molecules in purified MCs populations. Although knowledge is still fragmented, a panorama emerges in which this enigmatic immune cell type seems to participate in the three phases of cancer immunoediting (elimination, equilibrium, and escape) [5]. MCs can be attracted to tumor-derived chemotactic molecules, such as bioactive lipids, which we propose as novel molecules involved in MC migration to the tumors. Once infiltrated, MCs can recruit immune cells that are able to fight against tumors, and thus, MCs could play an important role in the first phase of cancer immunoediting, which is elimination (also known as immunosurveillance). Since the equilibrium and escape phases are fundamental for tumor cells, malignant cells need to overcome the immunosurveillance, and, in this regard, MCs seem to have relevance because they are also involved in processes that lead to tumor progression, such as angiogenesis, lymphangiogenesis, and metastasis. Regarding TME-induced phenotypic changes in MCs, we noticed that knowledge in that regard is scarce. We were able to find only two main studies that bring forward the hypothesis that MCs can be polarized in two distinct subsets: “MC1” anti-tumoral and “MC2” pro-tumoral phenotypes. Some key issues demand further investigation, such as the particular MC phenotypes that can be found in a single tumor, since distinct areas with particular conditions are found in the same tumor mass. In addition, it is necessary to identify the markers that characterize the possible MC phenotypes and the specific stimuli that lead to the polarization of MCs. We proposed that stimuli such as hypoxia could be crucial in the phenotype change of MCs and that studies addressing this question have to consider the fact that tumors are subjected to continuous changes in oxygen concentrations (cyclic hypoxia). Since continuous hypoxia induces the membrane translocation of secretory vesicles (as assumed by LAMP2 localization in the plasma membrane), it can be assumed that MCs acquire a particular secretory phenotype in those conditions. Evidence indicates that research on the relationship between MC activity and tumor growth should be intensified since that cell type emerges as a promising target against cancer, and the identification of pathways and stimuli that lead to pro-tumoral and anti-tumoral phenotypes could allow the “re-education” of MCs to promote their anti-tumor phenotype.

## Figures and Tables

**Table 1:** Localization and activity of MCs in common human malignant tumors and animal models of tumor growth.

Tumor Type	Mast Cell Activity/Function	Localization/Prognosis	References
Basal Cell Carcinoma (BCC) **	High numbers of MCs were found in BCC patients. MCs secrete IL-8 and RANTES to recruit lymphocytes and VEGF to promote angiogenesis.	Peritumoral	[14]
Breast Cancer **	MCs positive for estrogen receptor in the stroma of tumors are associated with low-grade tumors in BC patients.	Tumor stroma	[15]
	MCs may play a role in primary breast cancer angiogenesis. Infiltrated MCs may contribute to stromal remodeling and the α-SMA+ myofibroblast differentiation, both in BC patients.	Bad prognosis	[16,17]
Cholangiocarcinoma (ICC) **	Intratumoral MCs secrete histamine to promote angiogenesis, EMT of cancer cells, and ECM degradation in a mouse model. Cholangiocytes secrete Stem Cell Factor (SCF) to recruit MCs into the tumor in vitro.	Intratumoral	[18]
Colorectal Cancer (CRC) **	MCs secrete chymase to recruit macrophages, neutrophils, and other immune cells to improve host immunity against cancer in CRC patients.	Good prognosis	[19]
	Perivascular MCs promote angiogenesis and tumor progression at earlier and advanced stages. Peritumoral MCs positive for PAR-2 are associated with advanced CRC and numbers of MCs serve as a prognostic marker in CRC patients.	Peritumoral/Bad prognosis	[20,21,22]
Endometrial carcinoma	MCs have a preferential localization along blood vessels and sites of new vessel formation in human endometrial carcinoma samples.	Intratumoral/Bad prognosis	[23]
Gastric Cancer (GC) ** and Gastrointestinal Cancer (GIC) **	MCs promote angiogenesis and metastasis of cancer cells in GC patients.	Peritumoral/Bad prognosis	[24] and references therein
	In GC patients exist a positive correlation between MC numbers, IL-17 production and microvessel density, and numbers of neutrophils and regulatory Tregs.	Intratumoral/Bad prognosis	[25]
	MCs levels increase with tumor progression and predict reduced overall survival in GC patients.		[26]
Gynecologic Cancer **	High density of MCs in human samples of pre-malignant lesions of the cervix and endometrial cancer. MCs secrete tryptase to promote angiogenesis and invasion.	Peritumoral	[27]
	MCs are detected in ovarian cancer, uterine leiomyomas, vulva cancer, and the trophoblastic disease in women.	Peritumoral	[28]
Hepatocarcinoma (HCC) **	Peritumoral MC density positively correlates with the numbers of Tregs in HCC patients.	Peritumoral/Bad prognosis	[29]
	MCs secrete IL-17 to induce angiogenesis and tumor progression in HCC patients.	Peritumoral/Bad prognosis	[30]
Lung Cancer **	Intratumoral MCs indicate bad prognosis in human lung adenocarcinomas and advanced tumors.	Intratumoral/Bad prognosis	[31]
	Cytotoxic activity of TNF-α from MCs confers improved survival in NSCLC (non-small cell lung cancer) patients.	Intratumoral/Good prognosis	[32]
Melanoma **	Perivascular MCs secrete VEGF to promote angiogenesis, which correlates with malignancy and metastasis in a mouse model.	Peritumoral/Bad prognosis	[33] and references therein
	Low density of MCs indicates bad prognosis in human samples.	Peritumoral/Bad prognosis	[34]
Oral Squamous Cell Carcinoma	A higher MC density in human OSCC tumors is associated with a better prognosis.	Good prognosis	[35]
Pancreatic Cancer **	MCs secrete tryptase and IL-13 to promote cancer cells proliferation and invasion in vitro. Peritumoral MCs inter-communicate with pancreatic cancer cells by contact and high MC density indicates bad prognosis in a mouse model.	Peritumoral/Bad prognosis	[36,37]
Prostate Cancer	MCs secrete MMP-9 to promote angiogenesis and invasion and secrete high levels of FGF-2 in mouse and rat models.		[38,39]
	Metastasis is promoted by MCs via the regulation of the lncRNA-HOTAIR-PRC2-Androgen receptor-MMP9 signaling complex in human and mouse models.		[40]
	Peritumoral MCs are biomarkers at early stages of prostate tumor and indicate bad prognosis in humans.	Bad prognosis	[41] and references therein
	Intratumoral MCs are associated with a lower risk of prostate cancer recurrence, favorable tumor characteristics and good prognosis in PC patients.	Intratumoral/Good prognosis	[42,43]
Renal Cell Carcinoma	Intratumoral MCs promote tumor angiogenesis and acceleration of tumor growth in human and mouse models.	Bad prognosis	[44]
	MCs are associated with cell proliferation and recurrence in RCC patients.	Peritumoral/Bad prognosis	[45]
	The presence of intratumoral MCs in patients with RCC without metastasis after surgery functions as a predictive marker of survival and relapse.	Intratumoral/Good prognosis	[46,47]
Thyroid carcinoma	The secretion of histamine, CXCL1 and CXCL10 by human MCs promotes cancer cells’ proliferation, survival, and metastasis, as well as angiogenesis in vitro.	Bad prognosis	[48]

** Denotes high numbers of MCs.

**Table 2:** Tumor development in MC-deficient models.

**Sash Mice (c-kit W^sh^/W^sh^)** Mice bearing a large inversion (close to 70 kb) comprising the promoter of the *c-kit* gene (Wsh mutation). Constitutive lack of MCs, melanocytes, and interstitial cells of Cajal. Extramedullary myelopoiesis and systemic neutrophilia. Cardiac defects due to abnormal corin expression. Normal MCs numbers and neutrophils are reached after reconstitution with BMMCs from WT mice [49,50,51,52].	
**Tumor analyzed**	**Experimental approaches and main findings**
Melanoma	-Tumor generation: s.c. administration of 1 × 10^5^ B16.F10 cells above the right and left flanks. -Results: TLR-2 receptor agonist (Pam3CSK4) inhibited the growth of melanoma tumors, and this effect was attributed to MCs since it was not observed in MC-deficient mice. Tumor growth inhibition was restored in c-kit W^sh^/W^sh^ mice through local reconstitution with WT, but not with TLR2-deficient MCs [53].
	-Tumor generation: s.c. administration of 0.5 × 10^6^ B16.F1 melanoma cells into the left ear pinna. -Results: Monomeric IgE (mIgE) increased melanoma tumor growth, peritumoral MCs numbers, and blood vessels in WT but not in c-kit W^sh^/W^sh^ mice. Effects of mIgE on melanoma tumor growth were restored after reconstitution of sash mice with WT but not with Fyn ^−/−^ BMMCs [54].
	-Tumor generation: i.d. administration of B16-OVA melanoma cells and adoptive transfer of tumor-specific OT-I and OT-II T cells (TCs) was performed, followed by 3 consecutive peritumoral injections of LPS or vehicle. -Results: LPS-activated melanoma-resident MCs secreted CXCL10, which recruited tumor-infiltrating effector T cells (TILs) and started the immune response against melanoma. c-kit W^sh^/W^sh^ mice failed in inducing protective immune activation upon LPS exposure. Reconstitution of the skin of c-kit W^sh^/W^sh^ mice with WT BMMCs completely restored LPS-induced melanoma immune control [55].
Prostate adenocarcinoma	-Tumor generation: s.c. administration of 2 × 10^6^ cells from the T1525 and T23 cell lines. -Results: MCs promoted prostate tumor growth through MMP-9 production. Reconstitution of c-kit W^sh^/W^sh^ with WT but not with MMP-9^−/−^ MCs was enough to restore tumor growth [38].
Intestinal tumor	-Utilized cancer model: Murine model of multiple intestinal neoplasia (Min, APC^Min/*+*^). -Results: Sash mice developed 50% more adenomas, and the tumors were 33% larger than in their WT littermates. Eosinophils were fewer in adenomas from Min–Sash mice than in WT mice [56].
Pancreatic tumor	-Tumor generation: i.p. administration of 1 mg of tamoxifen in mice (pIns-mycERTAM; RIP7-bcl-xL), a murine model of Myc-induced β-cell carcinoma. -Results: Myc-induced pancreatic tumors were not observable on c-kit W^sh^/W^sh^ mice. MCs contributed to the generation of blood vessels and the macroscopic expansion of Myc-induced pancreatic islet tumors [57].
	-Utilized cancer model: Spontaneous mouse model of pancreatic ductal adenocarcinoma (PDAC) K-ras^G12V^. -Results: PDAC tumor growth was inhibited in c-kit W^sh^/W^sh^ mice, but it was observable in WT mice. Tumor development was recovered in sash mice reconstituted with WT BMMCs [37].
Bladder carcinoma	-Tumor induction: i.d. administration of 2.5 × 10^5^ MB49 cells. -Results: Sash female mice showed enhanced survival to MB49 tumors in comparison to WT female mice. Reconstitution of W^sh^ female mice with BMMCs diminished protective anti-tumor immunity [58].
Breast cancer	-Utilized cancer model: c-kit W^sh^/W^sh^ mice were crossed with mammary tumor model mice with the MMTV-Polyoma Middle T antigen (PyMT). -Results: c-kit W^sh^/W^sh^ mice showed delayed tumor growth, diminished tumor size, and reduced lung metastasis and angiogenesis compared to MC-proficient mice [59].
	-Utilized cancer model: Mammary tumor transgenic mouse strain MMTV-Polyoma middle T antigen (PyMT). -Results: MCs induced a luminal phenotype and modified the outcome of breast cancer tumors. The absence of MCs was associated with a lower grade of mammary tumors, with an evident reduction in metastasis dissemination [60].
Gastric cancer	-Utilized cancer model: c-kit W^sh^/W^sh^ mice were crossed with the gp130^F/F^ intestinal-type gastric cancer murine model. -Results: gp130^F/F^/c-kit W^sh^/W^sh^ mice showed significantly smaller and fewer tumors than their gp130^F/F^ littermates. MC deficiency diminished gp130^F/F^-driven tumor mass [61].
Squamous cell carcinoma	-Utilized cancer model: c-kit W^sh^/W^sh^ mice were crossed with E7 mice to obtain MC-deficient mice expressing the HPV16-E7 oncoprotein (E7.Kit W^sh^/W^sh^ mice). -Results: Whereas E7 mice showed tumor generation and no rejection of tumor grafts, E7 Kit W^sh^/W^sh^ mice showed increased rejection of skin grafts [62].
Malignant pleural effusion	-Utilized cancer model: malignant pleural effusions (MPEs) induced by LLC and MC38 adenocarcinomas. -Results: MPEs were not observed in c-kit W^sh^/W^sh^ mice, and the adoptive transfer of WT bone marrow, as well as MC reconstitution, restored MPEs in MC–deficient mice [63].
Lung adenocarcinoma (LADC)	-Utilized cancer model: LADC induced in KRAS^G12D^-transgenic mice. -Results: c-kit W^sh^/W^sh^ (KRAS^G12D^; c-kit W^sh^/W^sh^) showed protection from KRAS-driven alveolar carcinomas compared with KRAS^G12D^ mice. Tumor induction: i.d. injection of 10^6^ LLC cells into the rear flank dermis. -Results: c-kit W^sh^/W^sh^ mice displayed delayed primary tumor growth, as well as decreased spontaneous metastasis to the lungs compared with controls [64].
**Kit W^v^/Kit W^v^** Mice bearing a point mutation at the white spotting (W) locus together with a point mutation in the tyrosine kinase domain (Wv) of the protein CD117 (c-Kit). Expressed a truncated and inactive form of CD117. Constitutive lack of melanocytes, interstitial cells of Cajal, and MCs. Sterile, presented macrocytic anemia, gastric ulcers, and low numbers of bone marrow cells and circulating neutrophils. The very low number of MCs observed in these mice could be restored after reconstitution with BMMCs from WT mice [65,66,67].	
**Tumor analyzed**	**Experimental approaches and main findings**
Melanoma	-Tumor generation: s.c. administration of B16-BL6 melanoma cells (l0^5^ cells) in ear pinna. -Results: Kit W/W^v^ mice showed slower angiogenesis and a lower metastatic colony development rate than their WT littermates. Angiogenic response and incidence of hematogenous metastases were restored with WT bone-marrow reconstitution in Kit W/W^v^ mice [68].
Skin carcinogenesis	-Tumor generation: Administration of the carcinogen 7,12 dimethylbenz[a]-anthracene and subsequent treatment with the tumor promoter 12-tetradecanoyl phorbol-13-acetate (PMA). -Results: Tumor development and growth were increased in Kit W/Kit W^v^ mice. Reconstitution by local adoptive transfer of MCs normalized tumor incidence and growth [69].
Squamous cell carcinoma	-Utilized cancer model: Transgenic mouse model of HPV-associated squamous cell carcinoma. -Results: Premalignant angiogenesis was abolished in Kit W/Kit W^wv^ HPV16 transgenic mice [70].
Intestinal tumor	-Tumor generation: Administration of 1,2 dimethylhydrazine (DMH). -Results: Compared to WT mice, Kit W/Kit W^-v^ mice developed fewer tumors after treatment with DMH. Adoptive transfer of WT bone marrow cells to W/W^v^ mice increased the susceptibility to the development of DMH-induced tumors [71].
Lewis Lung Carcinoma (LLC)	-Tumor generation: s.c. administration of 2 × 10^6^ tumor cells in the right flank. -Results: Tumor incidence was higher in MC-deficient mice (Kit W/W^v^ and Kit W^v^/+) than in WT animals [72].
Fibrosarcoma	-Tumor generation: s.c. administration of 10^4^ MC-B6-1 tumor cells in the right flank. -Results: Tumor incidence was higher in MC-deficient mice (W/W^V^ and in W^V^/+) than in WT animals [72].
**Cre-mediated mast cell eradication (Cre-Master)** Mice generated by placing Cre expression under the control of the *Cpa*3 gene promoter. Deletion of 28 nucleotides of the first exon of *Cpa*3 locus. Constitutive lack of MCs in peritoneum, skin, and intestine. An important reduction in spleen basophil function. Some MC-dependent reactions could be reconstituted with BMMCs from WT mice [73].	
**Tumor analyzed**	**Experimental approaches and main findings**
Melanoma	-Utilized cancer model: Spontaneous tumor melanoma Tg(GRM1)EPv. -Results: Median tumor onset was significantly earlier in GRM1/Cre-Master mice than in Tg(GRM1)EPv control mice [74].
Skin carcinogenesis	-Utilized cancer model: Topical administration of 25 mg of 7, 12-dimethylbenz(a) anthracene (DMBA) on the back skin of 8- to 10-wk-old female mice, and one week later, 7.5 mg of the tumor promoter 12-tetradecanoyl phorbol-13-acetate (PMA) was applied weekly for 20 wk. -Results: Tumorigenesis-associated vascularization was not impaired in Cre Master mice [75].
Lung adenocarcinoma (LADC)	-Utilized cancer model: Mice received 10 consecutive weekly i.p. injections of urethane, a carcinogen contained in tobacco (1 g/Kg). -Results: Cpa3.Cre mice were markedly protected from urethane-induced bronchial carcinomas in terms of tumor multiplicity, size, and cellular proliferation rate. Tumor generation: i.d. administration of 10^6^ LLC cells into the rear flank dermis. -Results: Cpa3.Cre mice displayed delayed primary tumor growth and decreased spontaneous metastasis to the lungs compared with controls [64].
Malignant pleural effusion	Utilized cancer model: Malignant pleural effusions (MPEs) induced by LLC and MC38 adenocarcinomas. -Results: MCs were required for MPEs since Cpa3^Cre/+^ mice were protected from adenocarcinoma-induced effusions [63].
**Mcpt5-Cre+ R-DTA** Mice created by the crossing of transgenic mice expressing Cre recombinase under the control of the MC protease-5 (*Mcpt-5*) promoter and DTR-floxed mice. Inducible reduction in skin, stomach, and peritoneal MCs, which normally express *Mcpt-5* [76,77].	
**Tumor analyzed**	**Experimental approaches and main findings**
Melanoma	-Tumor generation: i.v. or s.c. administration of 1 × 10^5^ B16.F10 cells into the tail vein or hip region of male mice. -Results: Reduced melanoma colonization in lungs from Mcpt5-Cre+ R-DTA+ vs. Mcpt5-Cre- R-DTA+ mice was observed [33].
	-Tumor generation: i.d. administration of B16-OVA melanoma cells and adoptive transfer of tumor-specific OT-I and OT-II T cells (TILs) was performed, followed by 3 consecutive peritumoral injections of LPS or vehicle. -Results: Mcpt5-cre+ R-DTA^fl/fl^ mice failed to initiate melanoma immune defense upon LPS exposure as WT mice did [55].
Squamous cell carcinoma	-Utilized cancer model: Transgenic mouse model of HPV-associated squamous cell carcinoma. -Results: Absence of MCs had no impact on HPV-Induced epithelial growth and neovascularization, no differences were observed between MC-proficient (R26DTA/DTA Cre-negative) and MC-deficient (R26DTA/DTA Mcpt5-Cre + R-DTA) K14-HPV16 transgenic mice [78].
**“Hello Kitty” *Cpa*3*-Cre; Mcl-1fl/fl* Tg (Cpa3-cre) 3Glli; B6;129-*Mcl*1tm3sjkJ** Mice created by a cross between transgenic mice expressing Cre under the control of Cpa3 promoter and *Mcl-1* floxed mice. Constitutive low numbers of MCs in lung, peritoneum, skin, and trachea. Reconstitution of skin due to passive anaphylactic reactions caused by intradermal injection of BMMCs from WT mice. Lower numbers of basophils [79].	
**Tumor analyzed**	**Experimental approaches and main findings**
Gastric cancer	-Utilized cancer model: Mice *Cpa3-Cre; Mcl-1fl/fl* were crossed with the mouse model gp130^F/F^ of intestinal-type gastric cancer. -Results: MC-deficient gp130^F/F^;*Cpa3-Cre; Mcl-1/fl/fl* mice had significantly reduced tumor mass compared to their MC-proficient controls. Reduced angiogenic vessel density in the tumors of gp130^F/F^; *Cpa3-Cre;Mcl1fl/fl* mice [61].
**SI/SI^d^** Mice presenting a mutation at the Sl locus, codifying for Stem Cell Factor (SCF). Constitutive lack of melanocytes, germ cells, and interstitial cells of Cajal and MCs. Animals could not be reconstituted with BMMCs from WT mice, but MCs could be restored after injection of SCF [65,80,81].	
**Tumor analyzed**	**Experimental approaches and main findings**
Melanoma	-Utilized cancer model: Systemic intra-arterial injection of B16-G3.26 melanoma cells. -Results: Tumor metastasis in mutant SI/SI^d^ mice was evaluated. Mutant Sl/SI^d^ mice were found to have a markedly lower incidence of ovarian metastasis compared to normal congenic mice [82].
**W^s^/W^s^** Rats presented a deletion on the white locus (*c-Kit* gene, Ws, white spotting mutation). As a consequence, four amino acids close to the tyrosine autophosphorylation site of CD117 were absent in the protein. Constitutive of MC deficiency and anemia [83,84,85].	
**Tumor analyzed**	**Experimental approaches and main findings**
Breast cancer	-Tumor generation: Administration of 50 mg/kg of the chemical carcinogen N-nitrosomethylurea (NMU). -Results: W^s^/W^s^ rats did not show signs of neoplasia after treatment, compared to WT rats [86].

s.c., subcutaneous; i.v., intravenous; i.d., intradermal; i.p., intraperitoneal.
